# Aptamer Based, Non-PCR, Non-Serological Detection of Chagas Disease Biomarkers in *Trypanosoma cruzi* Infected Mice

**DOI:** 10.1371/journal.pntd.0002650

**Published:** 2014-01-16

**Authors:** Rana Nagarkatti, Fernanda Fortes de Araujo, Charu Gupta, Alain Debrabant

**Affiliations:** Laboratory of Emerging Pathogens, Division of Emerging and Transfusion Transmitted Diseases, Center for Biologics Evaluation and Research, U. S. Food and Drug Administration, Bethesda, Maryland, United States of America; Harvard School of Public Health, United States of America

## Abstract

Chagas disease affects about 5 million people across the world. The etiological agent, the intracellular parasite *Trypanosoma cruzi (T. cruzi)*, can be diagnosed using microscopy, serology or PCR based assays. However, each of these methods has their limitations regarding sensitivity and specificity, and thus to complement these existing diagnostic methods, alternate assays need to be developed. It is well documented that several parasite proteins called *T. cruzi* Excreted Secreted Antigens (TESA), are released into the blood of an infected host. These circulating parasite antigens could thus be used as highly specific biomarkers of *T. cruzi* infection. In this study, we have demonstrated that, using a SELEx based approach, parasite specific ligands called aptamers, can be used to detect TESA in the plasma of *T. cruzi* infected mice. An Enzyme Linked Aptamer (ELA) assay, similar to ELISA, was developed using biotinylated aptamers to demonstrate that these RNA ligands could interact with parasite targets. Aptamer L44 (Apt-L44) showed significant and specific binding to TESA as well as *T. cruzi* trypomastigote extract and not to host proteins or proteins of *Leishmania donovani*, a related trypanosomatid parasite. Our result also demonstrated that the target of Apt-L44 is conserved in three different strains of *T. cruzi*. In mice infected with *T. cruzi*, Apt-L44 demonstrated a significantly higher level of binding compared to non-infected mice suggesting that it could detect a biomarker of *T. cruzi* infection. Additionally, Apt-L44 could detect these circulating biomarkers in both the acute phase, from 7 to 28 days post infection, and in the chronic phase, from 55 to 230 days post infection. Our results show that Apt-L44 could thus be used in a qualitative ELA assay to detect biomarkers of Chagas disease.

## Author Summary

Chagas disease, caused by the parasite *Trypanosoma cruzi*, is a major health concern for people living in Latin America. There are no vaccines to prevent this disease and only two drugs are prescribed for treatment. Current methods to diagnose patients are not always successful and thus new methods need to be developed. One approach to develop an alternate method is to detect proteins and metabolites that are secreted by parasites into the blood of infected individuals. We have utilized a selection based method to isolate ligands that bind to these secreted proteins. These ligands, called aptamers, have been used to develop an assay that can detect the circulating parasite targets in the plasma or serum of an infected host. In an animal model of Chagas disease, our assay can detect parasite biomarkers as early as seven days after infection and as late as 230 days post infection. As the laboratory instruments and procedures are similar to performing an ELISA, the aptamer assay reported here could be easily performed at diagnostic facilities. Further improvement in this assay can lead to a new quantitative diagnostic test for Chagas disease. A similar selection based approach could also be used to develop ligands for the detection of biomarkers in other diseases.

## Introduction

Chagas disease affects about 5 million people in Mexico, Central and South America and is caused by the parasite *Trypanosoma cruzi (T. cruzi)*. The parasite survives in the infected mammalian host as two distinct life cycle forms, trypomastigotes, which are terminally differentiated motile extracellular forms that are present in blood and amastigotes, the intracellular replicative form found in infected nucleated cells [1], [2]. After infection, parasitemia in the blood increases and trypomastigotes are easily detectable by microscopy and PCR. This phase, called the acute phase, is followed by the chronic phase when the number of parasites in the blood reduces significantly and becomes undetectable by microscopy. Due to the presence of the trypomastigotes in blood, detection of parasite DNA by methods such as PCR is considered to be a very sensitive method to confirm infection [3]. However, this method does not always give a positive result as trypomastigote numbers may fluctuate in blood and sometimes fall below the detection limit of PCR assays, especially during the chronic phase [4], [5]. Alternatively, indirect methods such as detection of anti-*T. cruzi* antibodies, generated when the host immune response kicks in to control infection, provides a reliable method for diagnosis of Chagas disease. However, like PCR, conventional serological assays that detect antibodies to whole parasite lysates or recombinant antigens also have their limitations. For example, detection of parasites soon after infection may not be possible i.e. in the window period, and cross reactivity with antigens from related parasites such as *Leishmania* can lead to misdiagnosis. Due to the lifelong persistence of anti- *T cruzi* antibodies, conventional serological assays cannot be used to assess efficacy of drug treatment [6]–[8]. To overcome these problems, new “non-conventional” serological tests based on flow cytometry have been developed [9], [10]. However, these assays rely on expensive equipment and reagents that limit their application to reference laboratories only [11].

Thus, there is a need to develop new non-serological non-PCR based assays to address the limitations of the current methods available for *T. cruzi* detection. For this purpose, assays that detect biomarkers of Chagas disease need to be developed. Biomarkers are defined as characteristics that can be objectively measured and evaluated as indicators of pathogenic processes [12], [13]. Typically, biomarker discovery is based on comparing physiological, cellular, proteomic, metabolomic or genetic profiles between diseased and non-diseased individuals to determine patterns of characteristics that are prevalent in one group over the other [14], [15]. Biomarker discovery studies reported for Chagas disease lead to the identification of characteristics of host origin, such as host proteins or immune markers, which were elevated in Chagas disease [16]–[19]. However, one cannot exclude the possibility that these host markers could also be modulated in conditions unrelated to *T. cruzi* infection and thus these biomarkers have limited specificity [20], [21]. In order to overcome the issues of specificity the detection of pathogen specific factors would be ideal biomarkers of *T. cruzi* infection.

*T. cruzi* is an intracellular parasite and like other intracellular pathogens, has been shown to secrete parasite proteins into the host milieu [22]–[24]. These *T. cruzi* excreted secreted antigens (TESA) can be obtained from the *in-vitro* culture supernatant of infected host cells and have been used for serological assays [25], [26]. Recently, using advanced proteomics methods, significant progress has been made in identifying proteins expressed and secreted by the metacyclic trypomastigotes in culture medium [27]–[29]. Assays to detect parasite proteins in patient sera or urine samples have been explored in the past [22]. However, since the advent of conventional serological tests this approach to diagnose Chagas disease has been neglected. The detection of these circulating antigens can demonstrate that live parasites are present in the host even if direct detection of trypomastigotes in blood is negative. Assays that detect parasite TESA proteins using specific ligands could be developed as alternative non-PCR non-serological assays to confirm *T. cruzi* infection.

Aptamers have been used as specific ligands for pathogen detection and concentration applications [30], [31]. Aptamers are small RNA or DNA ligands that bind to their target with high affinity and specificity that are developed using SELEx (Systematic Evolution of Ligands by Exponential enrichment) methods [32]. The specificity of aptamer binding is derived from their tertiary structure, which depends on their primary sequence and by hydrophobic and ionic interactions with their targets [33]. The SELEx process is based on iterative steps where a random aptamer library is allowed to interact with the target. The bound aptamers are washed to remove low affinity and non-specific binders. The remaining bound aptamers are then recovered and amplified for the next step. At the end of this iterative procedure the sequences that survive the selection pressure are cloned and evaluated for their binding properties, such as, affinity and specificity [33]. SELEx is a powerful tool to develop ligands for biomarker based assays as it can be performed with crude protein extracts without *a priori* knowledge of the target [12], [34].

In this paper we have used SELEx to generate RNA aptamers against the excreted secreted antigens of *T. cruzi*. These aptamers were used as parasite specific ligands to develop Enzyme Linked Aptamer (ELA) assays to detect biomarkers in *T. cruzi* infected mice plasma.

## Materials and Methods

### Ethics statement

All animals were used under the auspices of a protocol approved by the Center for Biologics Evaluation and Research Animal Care and Use Committee (Protocol ASP # 2010-03). All the animal experiments were performed following the National Institutes of Health, Institute/Center, Animal Care and Use Committees guidelines for mice experiments.

### Materials

Unless otherwise specified, chemicals were of reagent grade and purchased from Sigma Aldrich (St. Louis, MO). Durascribe kit, to produce serum stable aptamers was purchased from Epicenter Technologies. Biotin-11-ATP was purchased from Perkin Elmer, USA.

### Parasite culture, total protein extract preparation, and collection of *T. cruzi* excreted secreted antigens (TESA)

*T. cruzi* parasites, Tulahuen, Y2, and a clinical isolate, 0704, obtained by hemoculture from a seropositive blood donor were cultured and maintained as described previously [30]. The epimastigotes for *T. cruzi* Y2 strain were cultured in LIT medium [35]. For specificity studies *Leishmania donovani* (*L. donovani*) promastigotes and axenic amastigotes were cultured as described before [36], [37]. To obtain the TESA fraction, *T. cruzi* trypomastigotes (Tulahuen and 0704) were allowed to infect a monolayer of murine 3T3 cells (obtained from American Type Culture Collection) and cultured in 5% fetal bovine serum (FBS) and Iscove's Modified Dulbecco's Media (IMDM, Invitrogen). After 24 hours of incubation, the monolayer was washed with medium to remove extracellular parasites. Fresh IMDM medium, without the FBS was added and cultures maintained over a period of 5 days. Supernatant was collected every 24 hours, centrifuged at 1500× g for 10 minutes, to remove trypomastigotes and cell debris, pooled and concentrated to 5 mg/ml total protein over a 30 kDa cutoff centricon filter (Millipore Inc.). This concentrated TESA preparation was used for all future experiments. Coomassie blue R250 staining of SDS PAGE was performed to compare protein profiles in infected vs. non infected cell culture supernatant (Figure S1). For whole parasite protein extract preparation, extracellular trypomastigotes and epimastigotes were collected from cultures, washed extensively with PBS and lyzed with 3 rounds of freeze thaw cycles. The lysate was further subjected to sonication with a Heat Systems (Ultrasonics Inc.) W-380 sonicator, set at 40% duty cycle, using 3 pulses of 30 seconds each on ice. Greater than 95% lysis of parasites was confirmed by microscopy. The lysate was then centrifuged at 16750× g for 60 minutes on a Beckman L8-80 M ultracentrifuge, to pellet cell debris and membranes. The concentration of soluble proteins was estimated using Bradford reagent.

### Generation of TESA specific RNA aptamers by SELEx

For the selection of RNA aptamers binding to parasite antigens, TESA preparation, diluted in PBS, was immobilized on a polystyrene 96 well MaxiSorp ELISA plate for 2 hours at room temperature. After immobilization the wells were washed 4 times with PBS to remove unbound proteins. The wells were then blocked with 1% bovine serum albumin (BSA) prepared in PBS. To prepare the initial RNA aptamer library, a pool of short DNA sequences, containing a central 80 nucleotide long randomized region, flanked by conserved T7 and SP6 polymerase binding sites was used in an *in-vitro* transcription reaction and RNA obtained purified over a G50 sephadex column (GE) [30]. Due to the random region, this RNA aptamer library was estimated to contain >10^12^ unique sequences. Nucleotide analogues 2′ F-dUTP and 2′ F-dCTP (fluorinated deoxynucleotides) were incorporated during the transcription reaction to make these RNA molecules resistant to serum nucleases [30], [38]. The RNA pool was denatured at 65°C for 10 minutes and then placed at room temperature for at least 30 minutes to refold the aptamers before performing SELEx. All subsequent procedures were then carried out at room temperature. The RNA aptamer pool was placed in uncoated wells to remove plastic binding molecules (Table S1). The aptamer solution was aspirated and then incubated in BSA coated wells to remove BSA binding aptamers (Table S1). These negative selection steps, with empty wells and BSA coated wells, were performed before each round of SELEx. The aptamer pool was recovered from the BSA coated wells and then incubated in TESA coated wells above. After binding to the TESA coated wells for varying times, the wells were washed 6 times with PBS (Table S1). During the final 3 rounds of the SELEx, wells were washed 12 times with PBS. At each round, the TESA bound aptamers were eluted using nuclease free water at 85°C for 10 minutes. RNA eluted from the TESA coated wells was reverse-transcribed using ThermoScript, a thermo stable reverse transcriptase and the cDNA was amplified using PCR conditions described before [30]. The PCR cycles were limited to 20 to prevent over amplification. The PCR product was desalted over a sephadex G50 spin column and used for *in-vitro* transcription reaction to generate RNA aptamer pool for subsequent rounds of SELEx. To prevent non-specific interactions between the RNA aptamer pool and immobilized TESA, all the SELEx rounds were performed in the presence of tRNA and salmon sperm DNA at a final concentration of 0.1 mg/ml each (Table S1).

To further prevent non-specific interaction of the RNA aptamer pool with host plasma proteins, negative selection was performed using wells coated with non-infected mouse plasma. After seven rounds of TESA binding the aptamer pool was bound to wells coated with *T. cruzi* infected mice plasma. Briefly, plasma obtained from *T. cruzi* Colombiana infected mouse was coated on ELISA plates at 1∶50 dilution and incubated for 2 hours. The wells were washed with PBS and aptamer pool was incubated for specified period (Table S1). A subsequent round of binding was done with TESA coated wells. This toggle SELEx, between wells coated with TESA in round 7 and wells coated with infected mouse plasma in round 8, was performed twice to enrich aptamers that bind to TESA protein present in infected mouse plasma (Table S1). After 10 rounds of SELEx the PCR product was cloned in pCR2.1 TOPO vector and 50 clones sequenced using standard ABI sequencing methods. Sequences obtained were analyzed using CLC Sequence Viewer and Sequencher 2.1. Phylogenetic analysis was performed to identify families of converged sequences from the aptamer pool. One individual member of each family was selected for further experiments. The SP6 forward and T7 reverse primers were used to PCR amplify the selected cloned sequences as described previously [30]. The PCR products obtained were ethanol precipitated and purified over a G50 sephadex column. Monoclonal RNA aptamers were then produced by *in-vitro* transcription of these PCR products as described above.

### Binding experiments

Aptamers were labeled with Biotin using biotin-11-ATP in the *in-vitro* transcription reaction as described before [30]. This resulted in the incorporation of approximately 1 biotin per aptamer. The binding assays were performed in a polystyrene 96 well ELISA plate. TESA, trypomastigote extract, epimastigote extract and other nonspecific proteins were bound to a 96 well polystyrene plate using 50 µl/well, at a concentration of 100 ng/µl, for 1 hour at room temperature. After the binding step, 1% BSA in PBS was used to block the nonspecific sites on the plate. Aptamers, diluted in 0.1% BSA in PBS at different concentrations, were added to the blocked wells and the plate incubated for 1 hour at room temperature. After this incubation step the plate was washed with PBS 3 times and then 50 µl Streptavidin-Alkaline phosphatase, diluted to 1∶2500 in 0.1% BSA prepared in PBS, was added to each well and the plate incubated for 30 minutes at room temperature. After 3 washes with PBS the wells were incubated with 4-Methylumbelliferyl phosphate, a fluorescent substrate for alkaline phosphatase. After one hour of incubation at 37°C, fluorescence was read by exciting at 340 nm and emission was recorded at 460 nm. For binding experiments with infected and non-infected mice plasma, plasma diluted at 1∶200 in PBS, was coated onto 96 well polystyrene plates. All other steps for this Enzyme Linked Aptamer (ELA) assay were identical to those described above. For competition experiments, infected mouse plasma was coated in a polystyrene 96 well plate at 1∶200 dilution prepared in PBS, incubated for 1 hour at room temperature and blocked with 1% BSA in PBS as described above. Refolded biotinylated Apt-L44 was incubated with various dilutions of TESA, trypomastigote extract, non-biotinylated Apt-L44 or non-biotinylated Apt-L44Sc, for 30 minutes on ice before applying to the blocked wells. After 1 hour incubation in the plasma coated wells, the plate was washed and developed with streptavidin alkaline phosphatase as described above. Control wells with biotinylated Apt-L44 alone or PBS alone were used to calculate percentage inhibition. PBS alone wells were considered as the background and the signal obtained was subtracted from all the wells. Biotinylated Apt-L44 alone wells were considered to be 100% and percentage inhibition was calculated using the formula for protein [(RFU ^aptamer+protein^/RFU ^aptamer alone^)×100] and for aptamer competition [(RFU ^aptamer+non-biotinylated aptamer^/RFU ^aptamer alone^)×100]. Data was plotted using GraphPad PRISM.

### Mice and parasite infection

Female C57BL/6 mice were purchased from NIH-NCI and Swiss Webster female mice were purchased from Taconic Inc. Mice were housed in cages under specific pathogen-free conditions and infected at 5–7 weeks of age. All animals were used under the auspices of a protocol approved by the Center for Biologics Evaluation and Research Animal Care and Use Committee. The myotropic Colombiana strain of *T. cruzi*, obtained from Dr. Ester Roffe (Laboratory of Molecular Immunology, National Institute of Allergy and Infectious Diseases, National Institutes of Health), was maintained by serial passage in Swiss Webster mice every 21 days [39]. They were infected intraperitoneally with 5000 blood stage trypomastigotes forms. C57BL/6 mice were infected intraperitoneally with 1000 trypomastigotes of *T. cruzi* isolated from the blood of infected Swiss mice. Parasitemia levels were determined by light microscopy by counting the number of parasites in an unstained 5 µl drop of whole blood drawn from the tail vein and mounted between a microscope slide and cover slip. The number of parasites was estimated as described in the literature [40]. Data was plotted and statistical analysis carried out using GraphPad PRISM. Unpaired t-test with Welch correction was performed with a 95% confidence interval and p-values calculated.

## Results and Discussion

### Selection of RNA ligands (aptamers) against *T. cruzi* excreted secreted antigens (TESA)

It has been reported previously that intracellular parasites such as *T. cruzi* excrete and secrete various proteins into the host milieu, both as free soluble proteins or contained in vesicles shed from the parasite cell surface [26], [41]. Detection of these parasite proteins in biological samples such as serum, plasma or urine, would indicate that a mammalian host is infected and thus provide information regarding *T. cruzi* pathogenesis [22], [42], [43]. For this purpose, we developed aptamers, RNA ligands that bind to *T. cruzi* excreted secreted antigens (TESA). TESA was prepared by concentrating 200 ml each, of 3T3 infected and non-infected FBS free cell culture medium, over a 30 kDa centricon filter. As the amount of protein from non-infected cells was found to be low, equal volumes from the concentrated preparations of infected and non-infected cell supernatant were run on a SDS PAGE gel and compared (Figure S1). More proteins were present in the infected culture supernatant compared to the non-infected culture supernatant. The complex mixtures of proteins in TESA were found to be predominantly in the high molecular weight range as shown previously [26], [44]. This concentrated TESA preparation was then used to perform SELEx. The RNA aptamer pool at round one was refolded and incubated with TESA proteins coated on ELISA plates as described in the methods section. RNA aptamers that remained bound to the TESA coated wells, after several washes with PBS, were recovered by denaturing them with nuclease free water at 85°C. The recovered aptamers were amplified by RT-PCR to prepare the library for the next round of selection. Iterative rounds of binding, washing and recovery were performed, for 10 rounds as described in the Method section and in Table S1.

During these iterative rounds of SELEx, selective pressure on the aptamer pools was increased by pre-incubating the aptamer pool with non-specific proteins such as BSA and by increasing the number of washes given after binding (Table S1). Additionally, as the goal was to detect parasite proteins in plasma or serum of infected mice, the aptamer pools, at rounds 8 and 10 were incubated with plasma from non-infected mice to remove all aptamers that could bind to host proteins. After removal of non-specific binders, the aptamer pool was subsequently incubated with plasma from infected mice to select aptamers that recognize and bind to circulating parasite antigens in the presence of anti-*T. cruzi* antibodies (Table S1).

### Isolation of aptamers that bind to TESA

**Figure 1:**
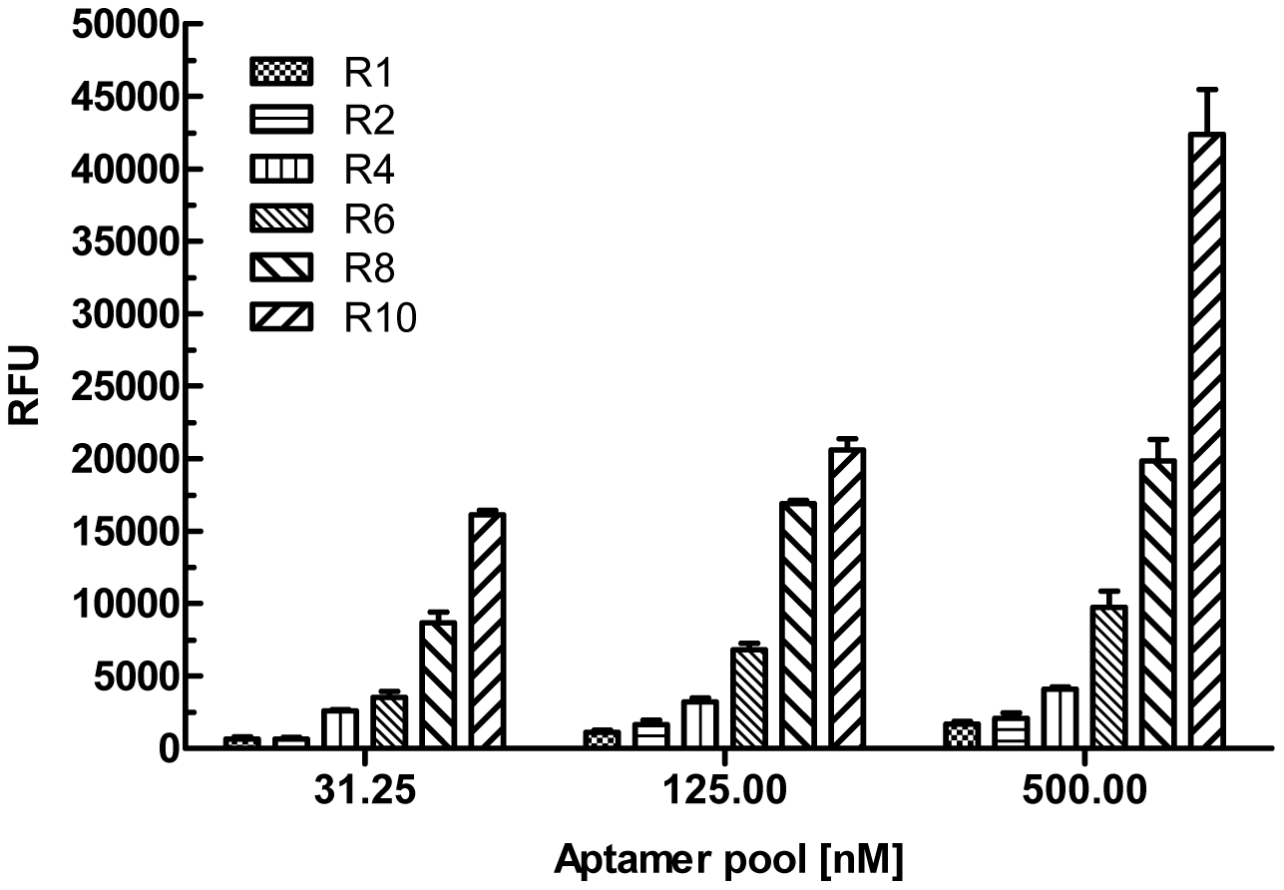
Binding analysis of aptamer pools at various rounds of TESA SELEx. Biotinylated aptamer pools obtained at rounds 1, 2, 4, 6, 8 and 10 of the TESA SELEx were used at various concentrations (31.25, 125 and 500 nM) in an ELA assay to demonstrate binding to TESA coated on a polystyrene 96 well plate. Signal generated, relative fluorescence units (RFU), are plotted on the Y-axis and each bar represents the mean of duplicate values with standard deviation. Data shows a gradual enrichment and evolution of TESA binding aptamers from the starting SELEx library to round 10.

**Figure 2:**
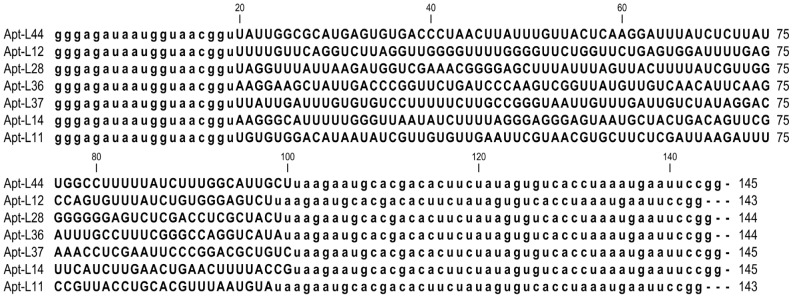
Sequence of TESA binding aptamers. RNA sequence, including the conserved T7 and SP6 primer binding sites (depicted in lower case) for the selected aptamers, Apt-L44, L12, L28, L36, L37, L14 and L11 obtained from round 10 of TESA SELEx, are represented. The nucleotide length of each aptamer is indicated on the right.

To determine if there is an increase in the proportion of aptamers that bind to TESA, from different rounds of SELEx, aptamer pools were biotinylated during the *in-vitro* transcription reaction using Biotin-11-ATP. Biotinylated aptamer pools, from rounds 1, 2, 4, 6, 8 and 10, were used to perform an Enzyme Linked Aptamer (ELA) assay with TESA coated at 2.5 µg/well (50 µl/well of a 50 ng/µl dilution) on ELISA plates as described in the methods section. The biotinylated aptamer pools were used in an assay similar to ELISA, except that instead of using antibodies, as in a conventional ELISA, the biotinylated aptamers were used to detect coated target antigens (Figure S2). After several washes to remove excess unbound aptamers, streptavidin-alkaline phosphatase conjugate was used to detect the amount of bound biotinylated aptamers and the plate developed using 4-MUP (Figure S2). The binding data indicated that indeed, as the SELEx progressed, the proportion of TESA binding aptamers that constitute the pools from rounds 1 to 10 increased significantly (Figure 1). Biotinylated aptamer pools at rounds 1, 2, 4, 6, 8 and 10 of the TESA SELEx was used at various concentrations (31.25, 125 and 500 nM) in an ELA assay to demonstrate binding to TESA coated on a polystyrene ELISA plate. For the concentration of 500 nM aptamer pool, compared to round 1 that showed an average signal of 1669.25 (±204.5) RFU, round 10 pool showed a signal 42365.71 (±3101.25) RFU (Figure 1). This represents a 25 fold improvement in signal indicating that TESA binding aptamers were enriched in round 10. This gradual enrichment and evolution of TESA binding aptamers from the starting SELEx library to round 10 can be observed in the increasing signal generated from the binding of the intermediate 2, 4, 6, 8 rounds as well (Figure 1). To identify individual aptamer clones present in the library, the round 10 aptamer pool was cloned into pCR-Topo 2.1 vector and sequenced. Phylogenetic analysis of 50 sequences cloned at round 10 of SELEx shows that the sequences had converged into 7 families (Figure S3). The converged sequences represented 73.4% of the total number of clones sequenced. Individual clones of each family were 95–100% identical to the other members of that family. A group of 7 individual plasmids, representing each family, were used to generate a set of monoclonal aptamers, Apt-L11, L12, L14, L28, L36, L37 and L44, for further binding analysis (Figure 2).

### Characterization of TESA binding aptamers

To perform binding assays with TESA and parasite protein extracts, individual aptamers were biotinylated during *in-vitro* transcription reaction using Biotin-11-ATP. This resulted in the incorporation of at least one biotin molecule per aptamer [30]. The biotin is linked to ATP via a 11 carbon linker and this prevents the biotin from interfering in the folding of the RNA aptamers [30]. These biotinylated aptamers were used in an ELA assay as described above (Figure S2). All 7 monoclonal aptamers obtained from round 10 pool were tested in a dose response experiment and showed significant binding to TESA (data not shown). As all the selected aptamers originated from round 10 pool that was bound to TESA we choose Apt-L44 as a model aptamer to further characterize the interaction of this ligand with parasite targets.

**Figure 3:**
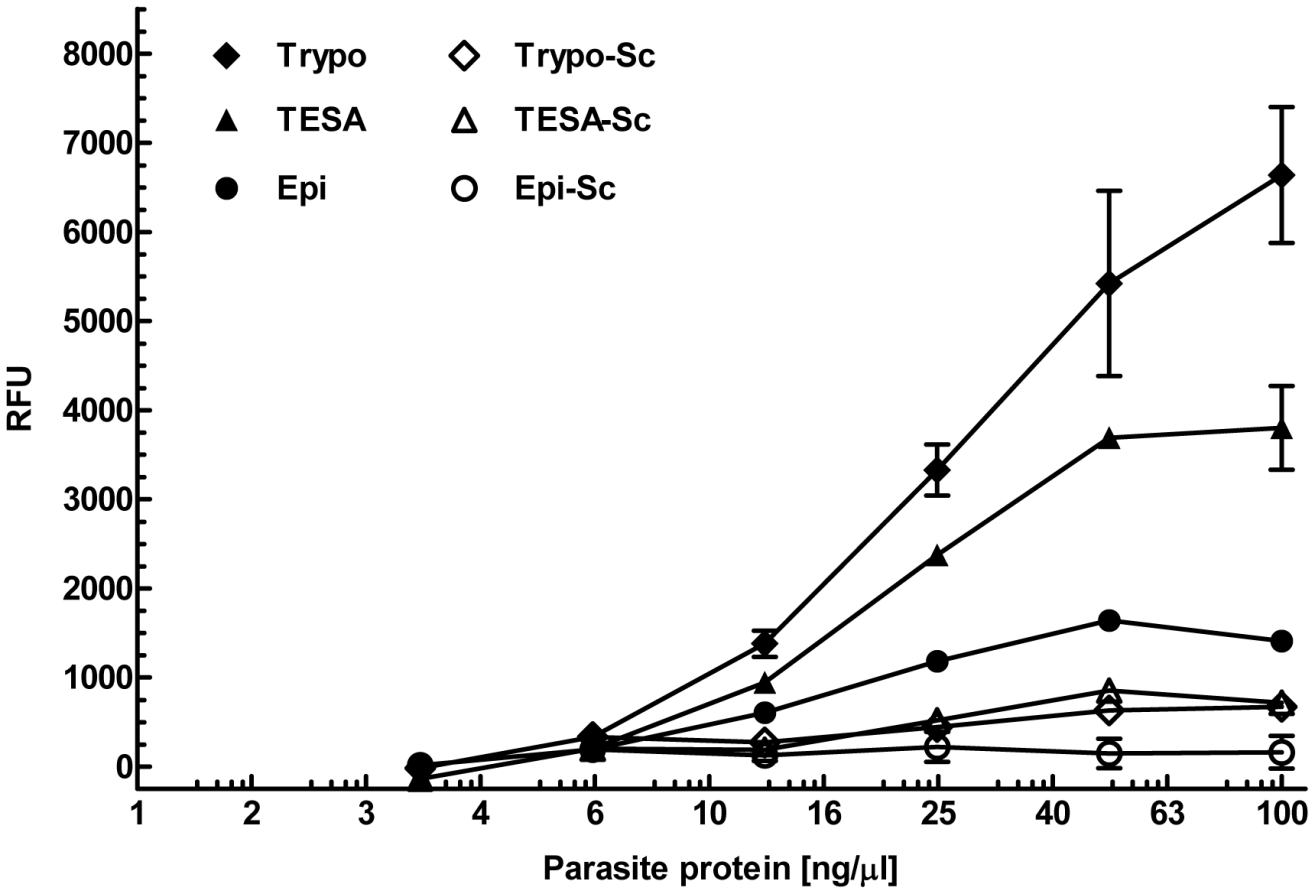
Aptamer L44 binds predominantly to TESA and protein extracts from *T. cruzi* trypomastigotes. 100-L44 was used to perform an ELA assay in a polystyrene 96 well plate coated with 50 µl/well of decreasing amounts, beginning at a concentration of 100 ng/µl, of TESA and trypomastigote extracts (Trypo) from *T. cruzi* Tulahuen strain, and epimastigote extracts (Epi) from *T. cruzi* Y2 strain. Signal generated, relative fluorescence units (RFU), are plotted on the Y-axis and each point represents the mean of duplicate values with standard deviation. Biotinylated Apt-L44 showed significant binding to TESA and trypomastigote protein extracts while minimal binding was observed to epimastigote extract. As a control scrambled biotinylated Apt-L44 was used and did not show any binding to the parasite proteins (Trypo-Sc, TESA-Sc and Epi-Sc).

Total soluble protein extracts from trypomastigotes, collected from culture supernatant of infected 3T3 cells, and from axenically cultured epimastigotes, were prepared as described in the methods section. For binding assays, various concentrations of TESA and parasite protein extracts were coated on an ELISA plate and probed with biotinylated Apt-L44 or biotinylated scrambled aptamer (Apt-L44Sc) at a concentration of 100 nM. To demonstrate that the interaction between Apt-L44 and parasite proteins was specific, a control aptamer, with the same nucleotide composition as Apt-L44 but with the primary nucleotide sequence scrambled (Apt-L44Sc) was synthesized. Due to the primary sequence being scrambled, the control aptamer (Apt-L44Sc) does not fold into the same three dimensional structure as Apt-L44 as predicted by the CLC software (Figure S4). Results showed that control scrambled aptamer did not bind to the parasite proteins suggesting that the primary sequence and thus the structure of Apt-L44 was responsible for aptamer-protein interactions (Figure 3). On the other hand, biotinylated Apt-L44 showed significant binding to TESA as well as to *T. cruzi* trypomastigote extract (Figure 3). Extracellular trypomastigotes from culture supernatants were collected by centrifugation and washed extensively with PBS before being used to produce the total trypomastigote soluble protein extract. These PBS washes were performed to exclude any host proteins from contaminating the trypomastigote extract. Unlike extracts prepared from parasites, TESA was prepared from parasite infected host cell culture supernatant, and therefore could be contaminated with host proteins. Since proteins expressed by trypomastigotes are known to be excreted/secreted as part of the TESA component, high binding to trypomastigote extract observed in these experiments strongly suggest that Apt-L44 binds specifically to *T. cruzi* expressed targets and not to host proteins that may have been present in the TESA preparation. Further, biotinylated Apt-L44 showed minimal binding to the epimastigote extract suggesting that the target of Apt-L44 is primarily expressed by the mammalian life cycle stage of the parasite.

### Apt-L44 binds to a conserved *T. cruzi* target

**Figure 4:**
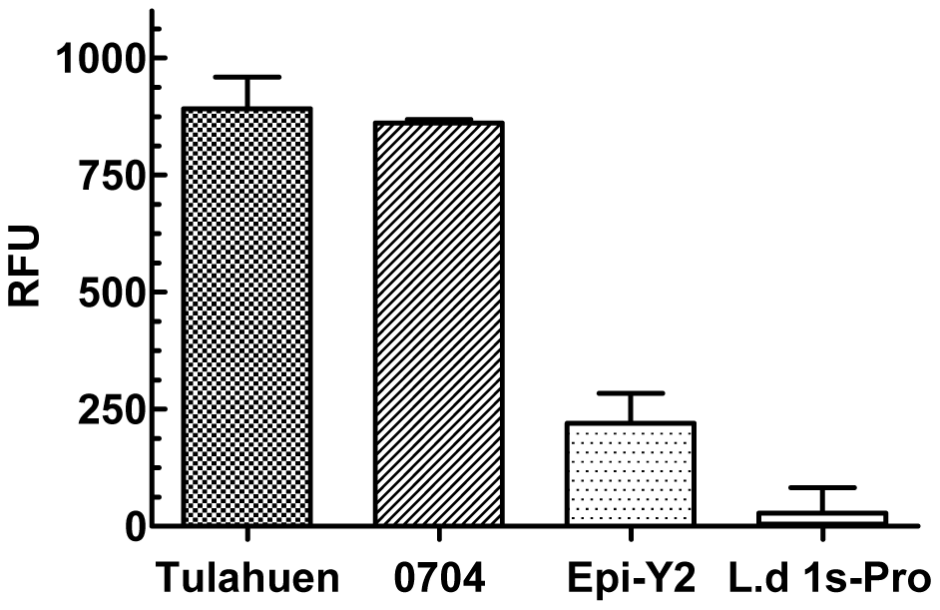
Aptamer L44 binds to a conserved target present in TESA from two strains of *T. cruzi*. Cell free culture supernatants, containing the excreted secreted antigens, from *T. cruzi* Tulahuen and 0704 strains were coated using 50 µl/well of 10 ng/µl dilution, and an ELA assay performed using biotinylated Apt-L44. As controls for specificity, culture supernatants, at 10 ng/µl, from axenic *T. cruzi* Y2 strain epimastigotes (Epi-Y2) and *L. donovani* 1 s promastigotes (L.d 1 s-Pro) were also used. Signal generated, relative fluorescence units (RFU), are plotted on the Y-axis and each point represents the mean of duplicate values with standard deviation.

To demonstrate the specific interaction between Apt-L44 and its target, cell free culture supernatants, from the *Tulahuen* and 0704 strains, were coated using 50 µl/well, at a concentration of 10 ng/µl, on a polystyrene 96 well plate and ELA assay performed. The signal from wells coated with only media were subtracted from the wells coated with parasite culture supernatants before analysis. Biotinylated Apt-L44 bound to TESA from both strains of the parasite suggesting that it binds to a target that is conserved between the two parasites strains (Figure 4). Minor binding to culture supernatant from epimastigotes was also observed. Although epimastigotes are the life cycle stage found in insects, they have the same genetic composition as the trypomastigotes, and thus it is conceivable that they express some TESA albeit at a lower level. However, culture supernatants from *L. donovani* promastigotes did not show any signal suggesting that the target of Apt-L44 may only be expressed by *T. cruzi* parasites. This result indicates the specific nature of aptamer interaction with its target and that this target is conserved between the two strains of *T. cruzi* tested (Figure 4).

### Apt-L44 can detect its target in plasma of *T. cruzi* infected mice

**Figure 5:**
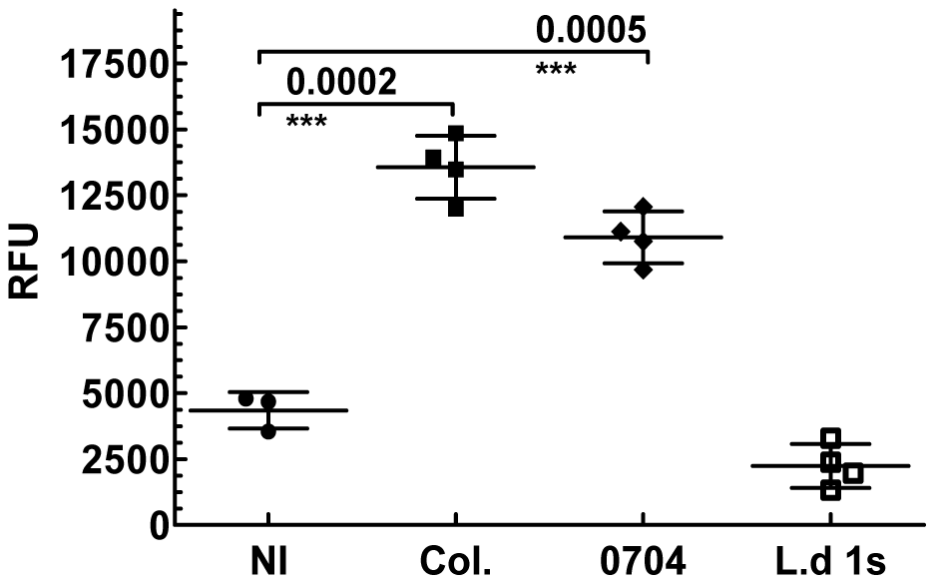
Apt-L44 ELA assay can detect mice infected with *T. cruzi*. Plasma, at 1∶200 dilution, from groups of mice infected either, the *T. cruzi* Colombiana (Col., n = 4) or, the 0704 (n = 4) strain, was coated on a polystyrene 96 well plate and ELA assay was performed using biotinylated Apt-L44. Signal generated, relative fluorescence units (RFU), are plotted on the Y-axis and each point represents the mean of duplicate values from individual mice. Group means and standard deviations are shown and statistical significance, compared with the non-infected group (NI, n = 4), determined using the unpaired t-test with Welch correction with a 95% confidence intravel. Biotinylated Apt-L44 showed significant binding to plasma from mice infected with either the Colombiana or the 0704 strains of *T. cruzi* and showed no binding with plasma from *L. donovani* infected mice (L.d 1 s, n = 4).

Once we established that Apt-L44 binds to a conserved target, expressed and secreted mostly by the blood stream form of the *T. cruzi* parasites, we wanted to determine if this target can also be detected in infected mouse plasma. For this, Swiss mice were infected with *T. cruzi* Colombiana strain or the 0704 strain and bled during the acute phase, 21 days post infection. A 1∶ 200 dilution of plasma prepared in PBS was coated on a polystyrene 96 well plate and ELA assay performed with 100 nM biotinylated Apt-L44. ELA assay results showed that biotinylated Apt-L44 bound to infected mouse samples at significantly higher levels than to the plasma from the control group (Figure 5). The aptamer bound to plasma of mice infected with either strain of parasites, further supporting earlier observation that Apt-L44 binds to a target conserved in both parasite strains. Taken together with culture supernatant binding data, Apt-L44 was shown to bind to its target expressed by three distinct parasite strains, the Tulahuen, Colombiana and 0704 strains (Figures 4 and 5). Apt-L44 binding to plasma from a control group of mice infected with *L. donovani* 1 s strain showed signal levels similar to the non-infected group suggesting that the interaction of aptamer was specific to a *T. cruzi* target (Figures 5). Although the progression of infection is different between *T. cruzi* and *L. donovani*, spleens obtained from *L. donovani* 1 s infected mice were significantly larger than spleens from non-infected control mice, suggesting that the parasite load was very high in the infected animals. These experiments indicate that indeed Apt-L44 can detect a biomarker of *T. cruzi* infection in mice.

### Apt-L44 biomarker is a component of TESA

**Figure 6:**
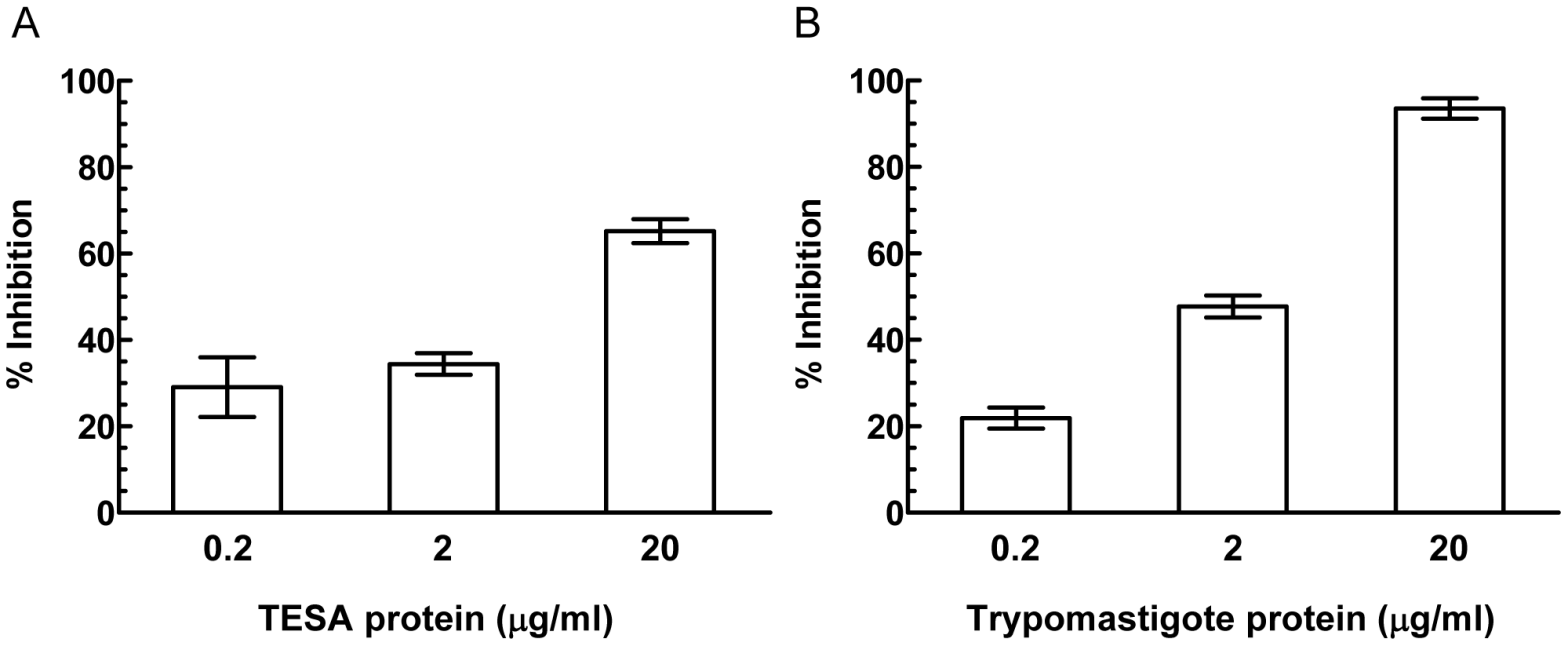
Competition with TESA and trypomastigote protein extract demonstrates that Apt-L44 binds to a parasite biomarker. Parasite protein were diluted at concentrations of 0.2, 2 and 20 µg/ml and incubated with 5 nM biotinylated Apt-L44. Plasma from infected mice, coated at 1∶200 dilution in a polystyrene 96 well plate, was then interrogated with the aptamer-protein mix and ELA assay performed. Wells with biotinylated Apt-L44 alone was considered 100% binding and percent inhibition calculated as described in the methods section. The percent inhibition was plotted on the Y-axis with each bar representing the mean % inhibition achieved, with standard deviation, from duplicate wells. (**A**) In the presence of TESA, biotinylated aptamer is sequestered by its target and cannot interact with the target present in the coated plasma, causing a decrease in signal levels represented by % inhibition. (**B**) Trypomastigote parasite proteins also showed a similar result as TESA and this suggested that Apt-L44 bound to a parasite specific biomarker circulating in the blood of *T. cruzi* infected mice.

**Figure 7:**
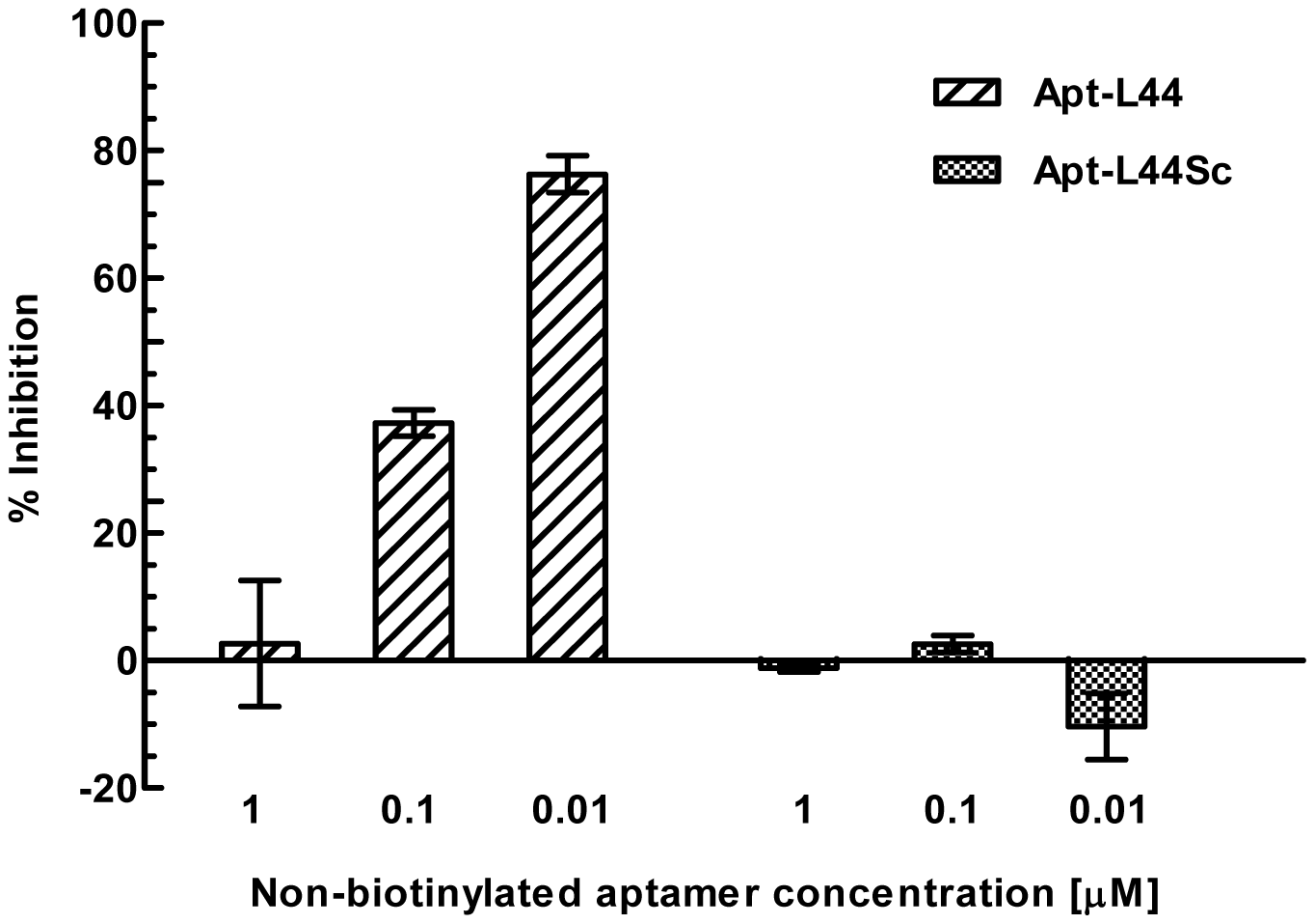
Competition with non-biotinylated Apt-L44 and Apt-L44Sc demonstrates that Apt-L44 interaction with its target is specific. Non-biotinylated aptamers, Apt-L44 (hashed bar) and control Apt-L44Sc (shaded bar) were diluted at various concentrations (1, 0.1 and 0.001 µM) and incubated with biotinylated Apt-L44. ELA assay was performed with 1∶200 dilution of mouse plasma infected with *T. cruzi* Colombiana. Wells with biotinylated Apt-L44 alone was considered 100% binding and inhibition calculated as described in the Methods. The percent inhibition was plotted on the Y-axis with each bar representing the mean % inhibition achieved, with standard deviation, from duplicate wells.

In order to further demonstrate that Apt-L44 detects the same target antigen in infected mice plasma, that is present in TESA or trypomastigote extracts, competition experiments were performed. Plasma from acutely infected mice was diluted 1∶200 in PBS and coated on 96 well polystyrene plates. Biotinylated Apt-L44 was incubated with serial dilutions of TESA or trypomastigote extracts for 30 minutes on ice. These aptamer solutions were then used in an ELA assay with the coated plasma samples. Signal from aptamer alone wells were considered to be 100% binding and percent inhibition was calculated using the formula [(RFU ^aptamer+TESA or trypomastigote extract^/RFU ^aptamer alone^)×100]. Both TESA and trypomastigote extracts were able to inhibit the binding of biotinylated Apt-L44 to the immobilized infected mouse plasma (Figure 6). This indicated that Apt-L44 target expressed by trypomastigotes, and found in the TESA, was also present in infected mouse plasma. Further, to prove that the inhibition was indeed due to quenching of biotinylated aptamer by the target, competition experiments were performed using non-biotinylated Apt-L44 and non-biotinylated scrambled aptamer. Biotinylated Apt-L44 was incubated with various dilutions of non-biotinylated, Apt-L44 or scrambled aptamer, for 30 minutes on ice. These aptamer pools were then used to perform ELA with infected mouse plasma. The biotinylated Apt-L44 alone was considered as 100% and inhibition calculated using the formula [(RFU ^aptamer+non-biotinylated aptamer (Apt-L44 or Scrambled)^/RFU ^aptamer alone^)×100]. Results showed that non-biotinylated Apt-L44 inhibited binding of biotinylated Apt-L44 to its target, while as expected, the scrambled aptamer showed no inhibition (Figure 7). From this experiment it was clear that indeed it was the Apt-L44 that bound to the parasite biomarker in the plasma of infected mice (Figure 7). These results are in agreement with those obtained before with TESA and trypomastigote extracts, where biotinylated scrambled aptamer did not bind to parasite proteins (Figure 3); further supporting the observation that the tertiary structure of Apt-L44 is critical and responsible for binding to its target. Together, results from these inhibition experiments, indicates that Apt-L44 could detect TESA in the plasma of an infected mouse. Apt-L44 ELA assay could thus be used as a qualitative assay to detect disease biomarkers in *T. cruzi* infected mice.

### Biomarker detection in acute and chronic *T. cruzi* mice infections

**Figure 8:**
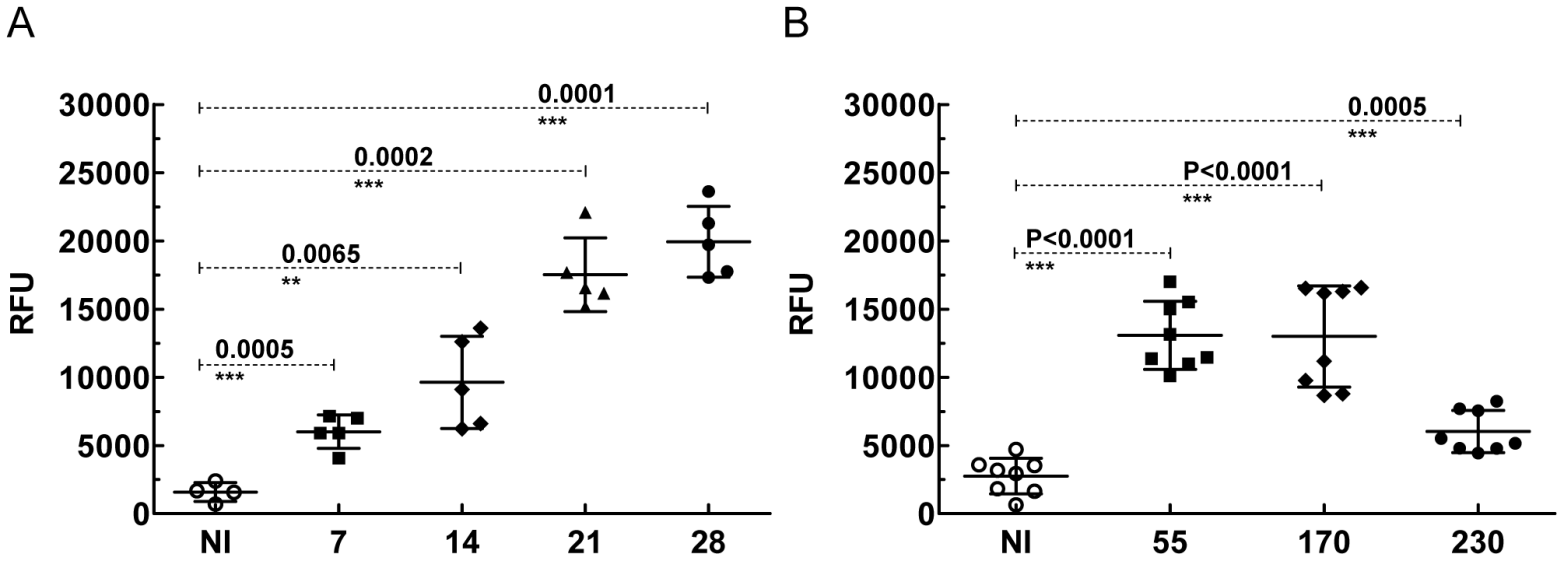
Apt-L44 detects levels of parasite biomarker circulating in the blood of *T. cruzi* infected mice. Plasma collected at various days from C57BL/6 mice infected with the Colombiana strain of *T. cruzi* was coated on a polystyrene 96 well plate and ELA assay performed using biotinylated Apt-L44. Signal generated, relative fluorescence units (RFU), are plotted on the Y-axis and groups of mice bled at various days post infection (dpi) represented on the X-axis. Each point represents the mean of duplicate values obtained from each individual mouse. Group means and standard deviations are shown and statistical significance, compared with the non-infected group (NI), determined using the unpaired t-test with Welch correction with a 95% confidence intravel. (**A**) The biomarker levels increased throughout the acute phase of the disease and there was a significant difference between the signal obtained from non-infected mice (n = 4) compared to the infected mice (n = 5) even as early as 7 days. (**B**) During the chronic phase, the biomarker levels were significantly higher in the infected groups (n = 8) compared to the non-infected group (n = 8).

In order to determine the levels of biomarker circulating in the blood of *T. cruzi* infected mice, studies with Apt-L44 were carried out with mice bled at regular intravels. A group of five C57BL/6 mice infected with *T. cruzi* Colombiana strain were bled at 7, 14, 21 and 28 days during the acute phase of infection and plasma tested with the Apt-L44 ELA assay. ELA assay was performed using 1∶200 dilution of the plasma, with 100 nM biotinylated Apt-L44. Results showed a significant difference in the signal obtained with infected mice, compared to the non-infected control mice, as early as 7 days post infection (dpi) (Figure 8A). The biomarker signal rose along with parasitemia in the blood and reached a peak at 28 dpi (Figure 8A). For the chronic phase, a group of 8 C57BL/6 mice infected with the Colombiana strain were bled at 55, 170 and 230 dpi, when parasites were undetectable by microscopy. ELA assay results showed a significant difference in the signal obtained from infected mice compared to the non-infected control mice at the three time points tested. The signals were similar at 55 and 170 dpi and lower at 230 dpi (Figure 8B). The half-life of the circulating biomarker, the level of expression of the biomarker by the parasite over time, or the overall parasite load in the infected animals, could explain the lower signal observed in the late chronic phase at 230 dpi. The relationship between these three factors is currently under investigation. These mouse studies, for acute and chronic phase, were repeated twice and in both cases similar observations were obtained. All the mice in the infected groups showed parasites by blood microscopy at the peak of acute phase; typically 25–30 days post infection. The fact that Apt-L44 gives a low signal with the non-infected mouse plasma indicates there is some non-specific interaction of the aptamer with host proteins. Attempts to reduce this non-specific interaction using detergents or high salt conditions have been unsuccessful. High detergent concentrations affect the stability of RNA secondary structure, thus reducing the overall signal in the assay. Due to the qualitative nature of this assay it is not possible to make a direct comparison to PCR or serology and additional studies are being conducted to develop this assay into a quantitative test.

In this study, we have successfully demonstrated the power of SELEx technology to develop pathogen specific ligands, RNA aptamers, to detect *T. cruzi* biomarkers in the blood of infected mice. The SELEx approach is extremely powerful in that, without the knowledge of what the biomarker is, i.e., its identity, we are still able to develop ligands that are pathogen specific. The ability to use complex mix of targets, such as TESA, suggests that this approach can be used to develop biomarker based assays for other intracellular and extracellular pathogens. Additional studies to improve this biomarker detection assay are being performed. Identifying the target of Apt-L44 using a proteomics approach will help in isolating additional aptamers against the recombinantly expressed protein. These aptamers can then be used in sandwich ELA assay to increase sensitivity and develop a quantitative assay. The second generation, quantitative ELA assays will be evaluated using human clinical specimens from Chagas endemic areas. These assays can also be used in drug discovery applications to assess drug efficacy *in-vivo* as serological assays are not useful for this purpose due to lifelong presence of parasite antibodies in treated animals.

## Conclusions

We have developed pathogen specific ligands, called aptamers, using the SELEx method. These aptamers were used in assays to detect *T. cruzi* biomarkers in infected animals. Current detection methods based on PCR demand a highly controlled environment free of potential contaminating DNA, expensive reagents and complex instrumentation. ELA assays can be performed using standard ELISA equipment. Conventional ELISA platforms are highly standardized with reagents and equipment readily available in most laboratories. The aptamer based assay can be used as an alternate non-PCR, non-serology based method to detect *T. cruzi* infection.

## Supporting Information

Figure S1 **SDS PAGE profile of** ***T. cruzi*** **excreted secreted antigens (TESA).** Culture supernatant from non-infected 3T3 cells (lane 1) and *T. cruzi* infected murine 3T3 adherent cells (lane 2) were concentrated over a 30 kDa centricon filter, separated on a 10% denaturing SDS PAGE gel and stained with coomassie 250 stain. The protein standards (kDa) run simultaneously have been marked. A large number of proteins, majority of which are of high molecular weight, are visible in the TESA fraction compared to the non-infected culture supernatant.
(TIF)

Figure S2 **Schematic representation of the Enzyme Linked Aptamer (ELA) assay.** Parasite protein extracts and TESA are coated on polystyrene 96 well plates and blocked with 1% BSA in PBS for 1 hour at room temperature. Biotinylated aptamer (or biotinylated aptamer pools) refolded and in their stable conformation at room temperature, are incubated with the blocked wells for 1 hour. After washing the wells with buffer, streptavidin-alkaline phosphatase is added to detect the bound biotinylated aptamer. The plate is then washed and incubated with a fluorescent alkaline phosphatase substrate, 4 methylumbelliferyl phosphate. After one hour of incubation at 37°C, the plate is read using a plate reader (excitation at 360 nm, emission at 440 nm, and cutoff filter at 435 nm). The signal generated is plotted as relative fluorescence units and data analyzed using GraphPad PRISM software.
(TIF)

Figure S3 **Phylogenetic analysis of sequences obtained from TESA SELEx.** Aptamer pool obtained at round 10 of the TESA SELEx was cloned into a TOPO cloning vector and 50 individual clones were isolated and sequenced. Aptamer sequences were analyzed using the Sequencher 2.4 software and aligned using the CLC Sequence Viewer 6.4 software. The Unweighted Pair Group Method using arithmetic averages (UPGMA) algorithm for distance data was employed to obtain converged families. Bootstrapping was performed with 1000 replicates and families obtained were labeled 1 thru 7. A single clone from each family was selected for TESA binding studies.
(TIF)

Figure S4 **Secondary structure of Apt-L44 and Apt-L44Sc.** (**A**) Nucleotide sequence including the conserved T7 and SP6 primer binding sites, depicted in lower case letters, of Apt-L44 and Apt-L44Sc are shown. (**B**) Predicted secondary structure obtained from Minimal Free Energy (MFE) calculations of Apt-L44 is shown with the shaded sequence representing the T7 and SP6 primer binding sites respectively. (**C**) Predicted secondary structure obtained from Minimal Free Energy (MFE) calculations of Apt-L44Sc. Gibbs free energy (ΔG) calculated for each aptamer is shown.
(TIF)

Table S1 **Conditions utilized for performing SELEx with TESA.** TESA SELEx was performed by coating the antigen on ELISA plates. For negative SELEx, refolded aptamer pools were added to empty wells to remove aptamers that can bind to polystyrene (PB) or to BSA coated wells alone (BB) for the amount of time indicated. The non-specific aptamer depleted supernatant, was then incubated with TESA coated wells that were blocked by BSA to recover TESA binding (TB) aptamers for the time indicated per round (R1 to R10). As the rounds progressed SELEx conditions were modified to prevent nonspecific interactions by including 0.1 mg/ml t-RNA, 0.1 mg/ml salmon sperm DNA (ssDNA) and 0.1% BSA. Additionally, negative SELEx, to remove aptamers in the library that could bind to host proteins, was carried out by coating normal mouse plasma on wells and incubating the aptamer pools in round 8 and 10. After incubation for a specified time, as shown in the table, the aptamer pool was aspirated and incubated with infected mouse plasma. To increase the selection pressure, the amount of aptamer RNA pool used during subsequent rounds were reduced from 500 pmol to 12.5 pmol and the number of washes increased from 1 to 6, 30 minute washes, as specified in the table.
(TIF)
