# Supplementary figures and images for: Aptamer Based, Non-PCR, Non-Serological Detection of Chagas Disease Biomarkers in Trypanosoma cruzi Infected Mice
Source: PLoS Negl Trop Dis. 2014 Jan 16;8(1):e2650. doi: 10.1371/journal.pntd.0002650 (PMC3894185; doi:10.1371/journal.pntd.0002650)

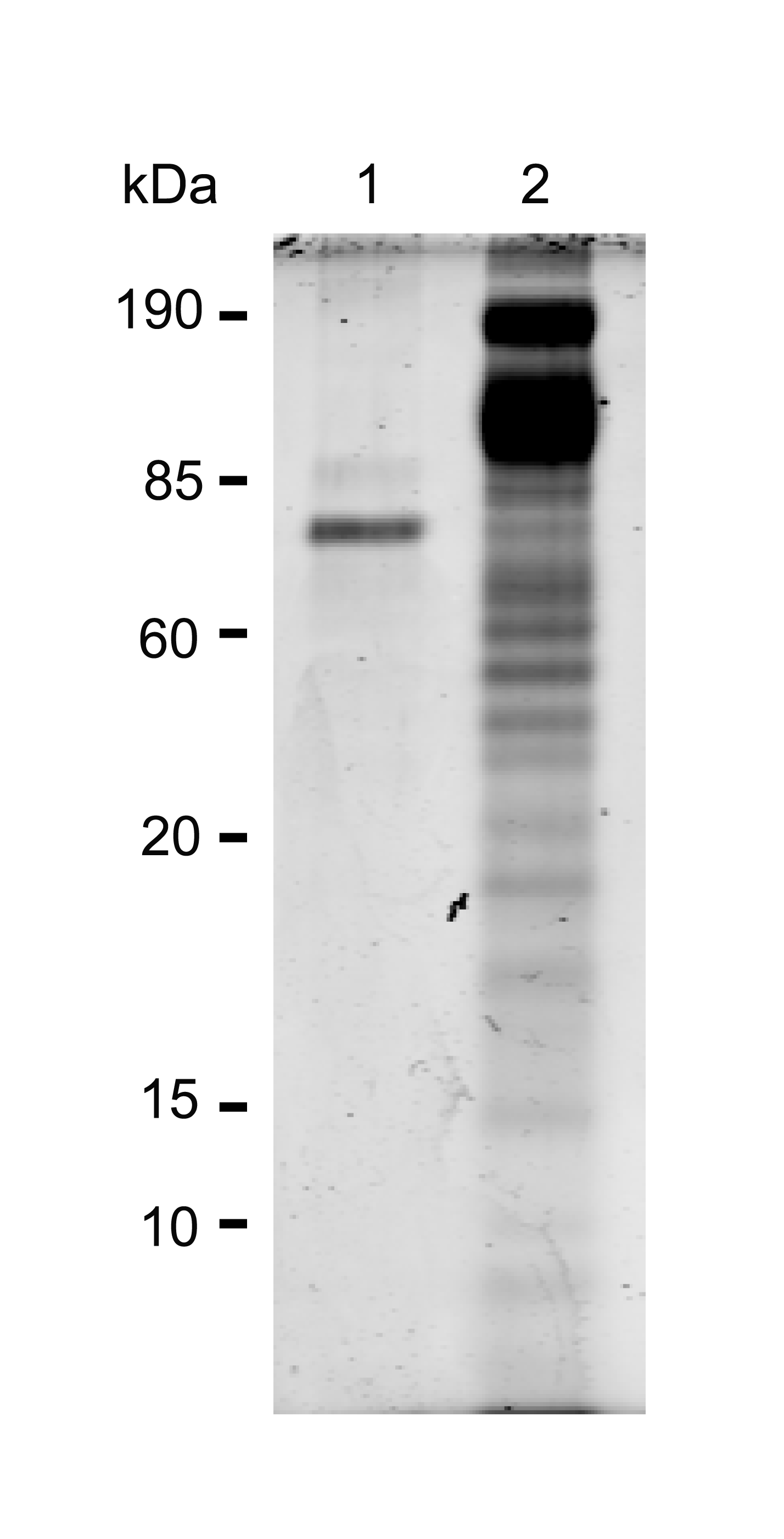

Supplement: Figure S1 — SDS PAGE profile of T. cruzi excreted secreted antigens (TESA). Culture supernatant from non-infected 3T3 cells (lane 1) and T. cruzi infected murine 3T3 adherent cells (lane 2) were concentrated over a 30 kDa centricon filter, separated on a 10% denaturing SDS PAGE gel and stained with coomassie 250 stain. The protein standards (kDa) run simultaneously have been marked. A large number of proteins, majority of which are of high molecular weight, are visible in the TESA fraction compared to the non-infected culture supernatant. (TIF) [file pntd.0002650.s001.tif]

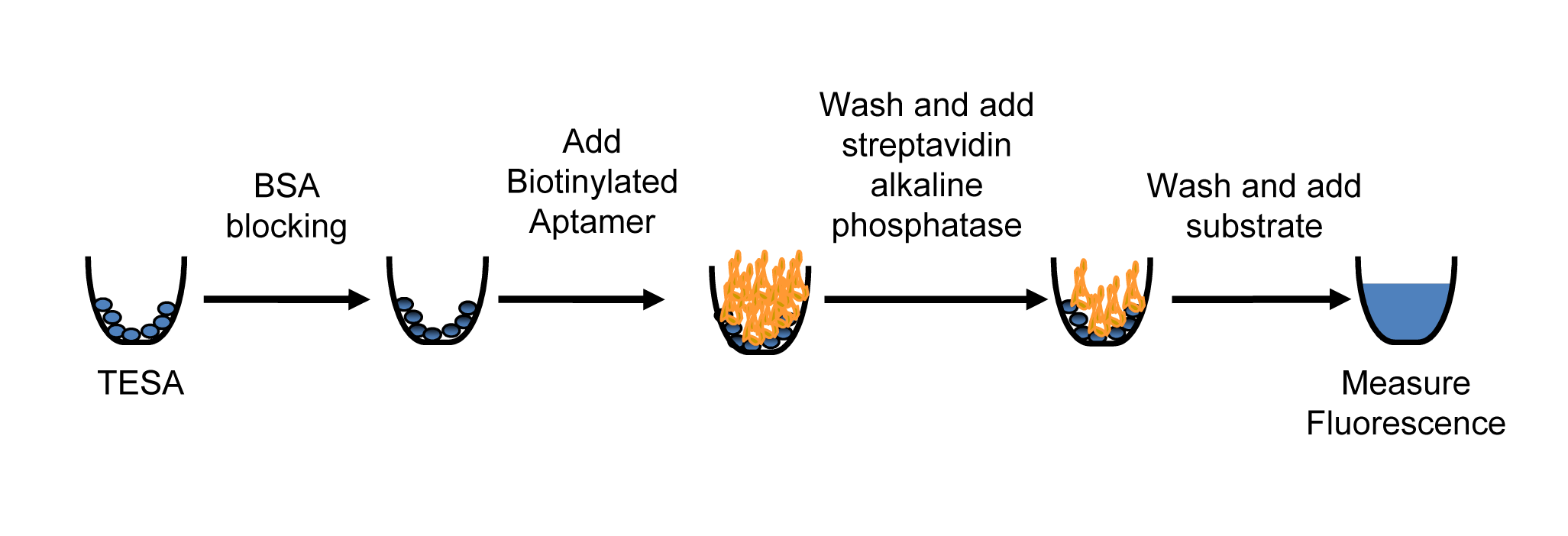

Supplement: Figure S2 — Schematic representation of the Enzyme Linked Aptamer (ELA) assay. Parasite protein extracts and TESA are coated on polystyrene 96 well plates and blocked with 1% BSA in PBS for 1 hour at room temperature. Biotinylated aptamer (or biotinylated aptamer pools) refolded and in their stable conformation at room temperature, are incubated with the blocked wells for 1 hour. After washing the wells with buffer, streptavidin-alkaline phosphatase is added to detect the bound biotinylated aptamer. The plate is then washed and incubated with a fluorescent alkaline phosphatase substrate, 4 methylumbelliferyl phosphate. After one hour of incubation at 37°C, the plate is read using a plate reader (excitation at 360 nm, emission at 440 nm, and cutoff filter at 435 nm). The signal generated is plotted as relative fluorescence units and data analyzed using GraphPad PRISM software. (TIF) [file pntd.0002650.s002.tif]

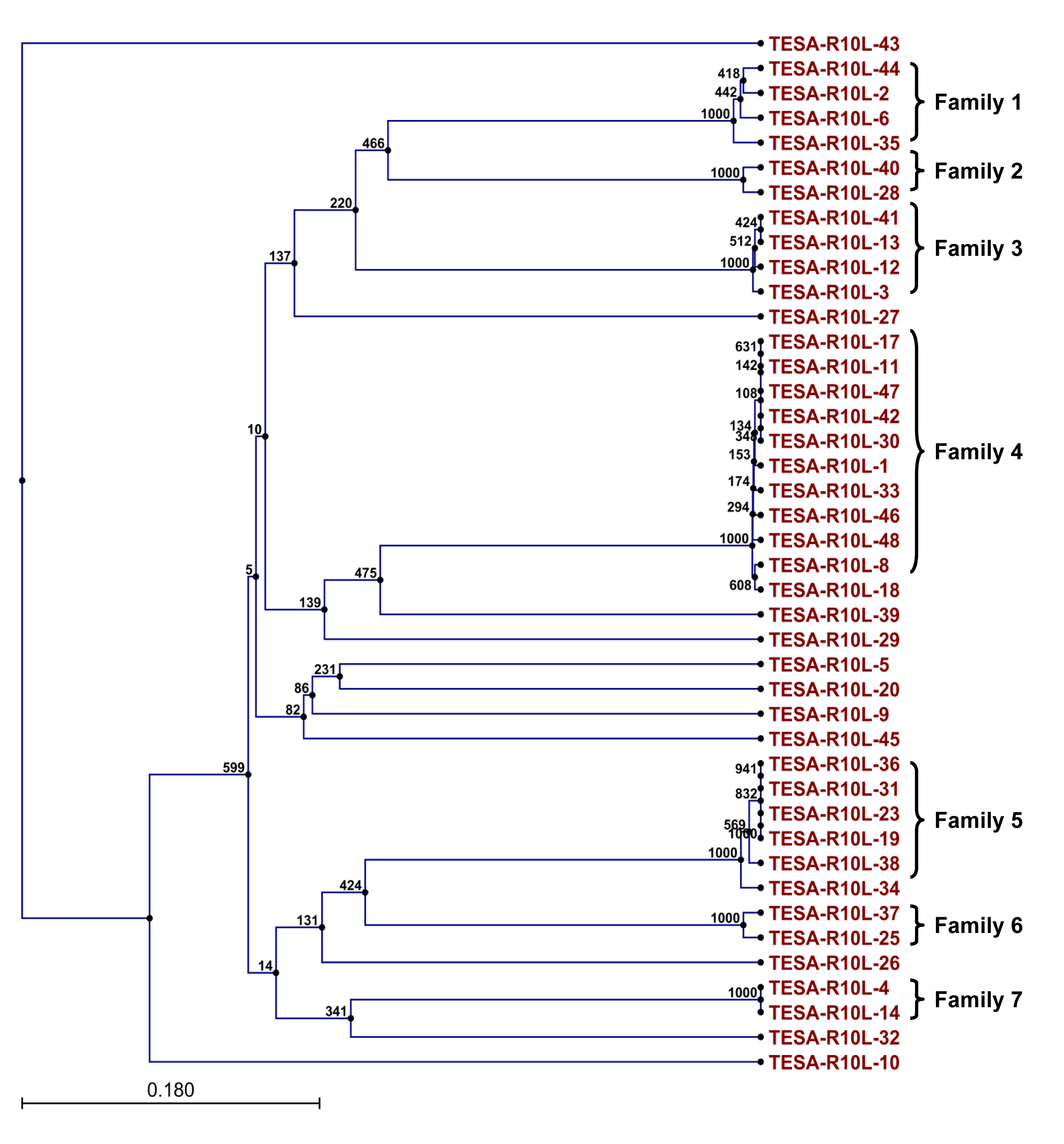

Supplement: Figure S3 — Phylogenetic analysis of sequences obtained from TESA SELEx. Aptamer pool obtained at round 10 of the TESA SELEx was cloned into a TOPO cloning vector and 50 individual clones were isolated and sequenced. Aptamer sequences were analyzed using the Sequencher 2.4 software and aligned using the CLC Sequence Viewer 6.4 software. The Unweighted Pair Group Method using arithmetic averages (UPGMA) algorithm for distance data was employed to obtain converged families. Bootstrapping was performed with 1000 replicates and families obtained were labeled 1 thru 7. A single clone from each family was selected for TESA binding studies. (TIF) [file pntd.0002650.s003.tif]

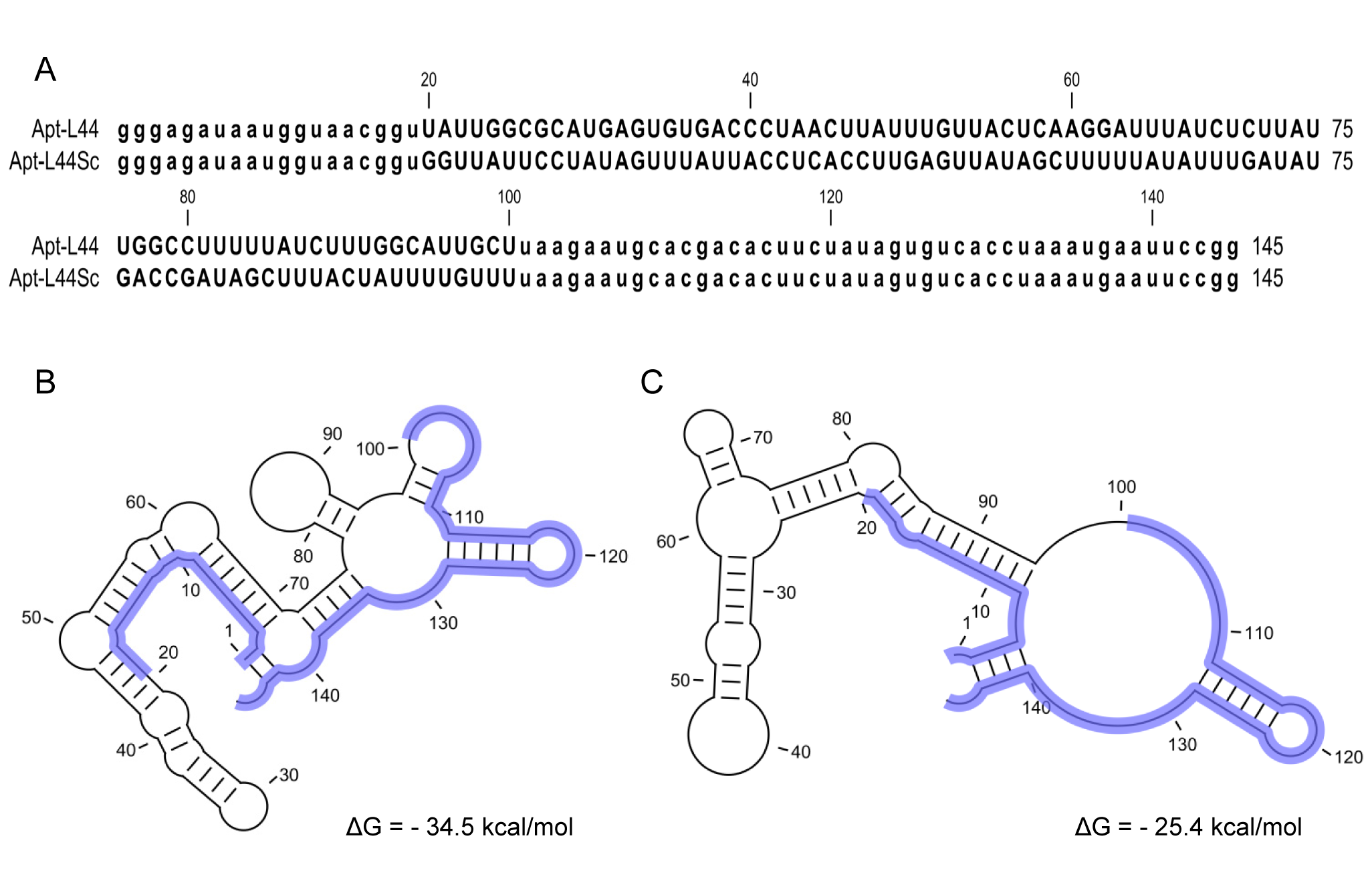

Supplement: Figure S4 — Secondary structure of Apt-L44 and Apt-L44Sc. (A) Nucleotide sequence including the conserved T7 and SP6 primer binding sites, depicted in lower case letters, of Apt-L44 and Apt-L44Sc are shown. (B) Predicted secondary structure obtained from Minimal Free Energy (MFE) calculations of Apt-L44 is shown with the shaded sequence representing the T7 and SP6 primer binding sites respectively. (C) Predicted secondary structure obtained from Minimal Free Energy (MFE) calculations of Apt-L44Sc. Gibbs free energy (ΔG) calculated for each aptamer is shown. (TIF) [file pntd.0002650.s004.tif]

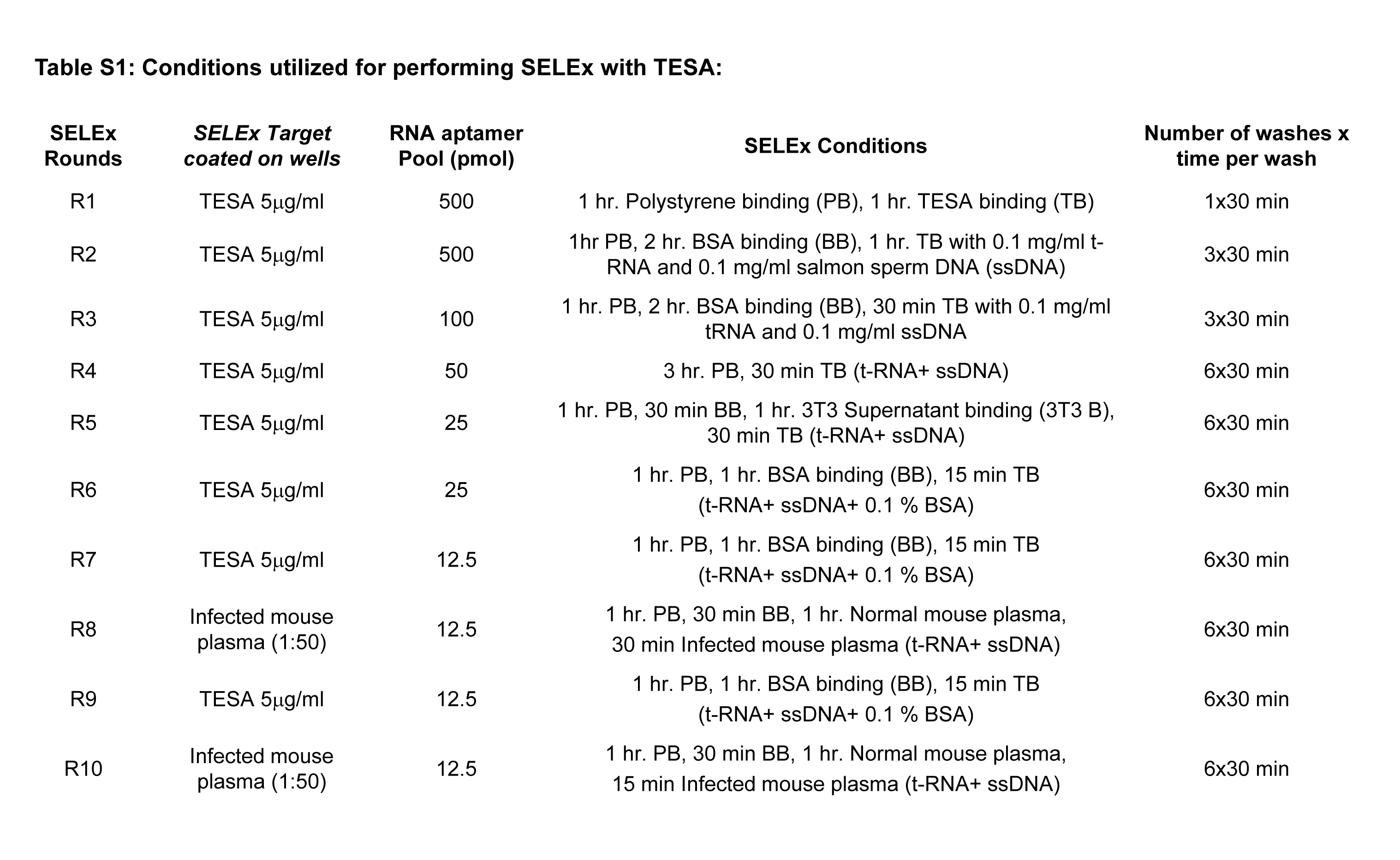

Supplement: Table S1 — Conditions utilized for performing SELEx with TESA. TESA SELEx was performed by coating the antigen on ELISA plates. For negative SELEx, refolded aptamer pools were added to empty wells to remove aptamers that can bind to polystyrene (PB) or to BSA coated wells alone (BB) for the amount of time indicated. The non-specific aptamer depleted supernatant, was then incubated with TESA coated wells that were blocked by BSA to recover TESA binding (TB) aptamers for the time indicated per round (R1 to R10). As the rounds progressed SELEx conditions were modified to prevent nonspecific interactions by including 0.1 mg/ml t-RNA, 0.1 mg/ml salmon sperm DNA (ssDNA) and 0.1% BSA. Additionally, negative SELEx, to remove aptamers in the library that could bind to host proteins, was carried out by coating normal mouse plasma on wells and incubating the aptamer pools in round 8 and 10. After incubation for a specified time, as shown in the table, the aptamer pool was aspirated and incubated with infected mouse plasma. To increase the selection pressure, the amount of aptamer RNA pool used during subsequent rounds were reduced from 500 pmol to 12.5 pmol and the number of washes increased from 1 to 6, 30 minute washes, as specified in the table. (TIF) [file pntd.0002650.s005.tif]
